# The use of neutrophil to lymphocyte ratio as a predictor for clinical outcomes in spontaneous intracerebral hemorrhage

**DOI:** 10.18632/oncotarget.20120

**Published:** 2017-08-10

**Authors:** Zengpanpan Ye, Xiaolin Ai, Fang Fang, Xin Hu, Andrew Faramand, Chao You

**Affiliations:** ^1^ Department of Neurosurgery, West China Hospital of Sichuan University, Chengdu, Sichuan, China; ^2^ Department of Neurosurgery, University of Pittsburgh Medical Center, Pittsburgh, Pennsylvania, United States of America

**Keywords:** neutrophil to lymphocyte ratio, intracerebral hemorrhage, meta-analysis

## Abstract

**Objective:**

Neutrophil to lymphocyte ratio (NLR) is used as an independent predictor for clinical outcomes in cancers, cardiovascular disorders and ischemic stroke. The prognostic role of NLR in spontaneous intracerebral hemorrhage (sICH) is still controversial. The aim of this report is to conduct a meta-analysis to evaluate the prognostic significance NLR in patients with sICH.

**Materials and Methods:**

All related articles were searched on PubMed, EMBASE, Cochrane Central Register of Controlled Trials followed the PRISMA flow diagram. The quality of eligible studies were evaluated and the related data were extracted by two reviewers independently. The end points included the mortality and poor outcomes and subgroup analyses were performed.

**Results:**

Five studies with 1944 subjects were included and had acceptable quality. The high NLR had a higher risk of in-hospital mortality (OR: 0.97; 95% CI: 0.94–0.99, *p* = 0.02) and 90-day mortality (OR: 2.43; 95% CI: 1.01–5.83, *p* = 0.047); without association with the poor outcomes (OR: 1.17; 95% CI: 0.93–1.47, *p* = 0.18). After subgroup analyses, the high NLR correlated with an increased 90-day mortality in the high cut-off group (OR: 1.56; 95% CI: 1.15–2.13, *p* = 0.005). The high NLR additionally predicts poor outcomes in smaller hematoma group (OR: 1.16; 95% CI: 1.01–1.32, *p* = 0.04) and the high cut-off group (OR: 2.20; 95% CI: 1.54–3.14, *p* < 0.001).

**Conclusions:**

The high NLR was significantly associated with in-hospital and 90-day mortality in patients with sICH. The NLR with cut-off of 7.5 had statistically significant potential for predicting mortality and poor outcomes, regardless of country, time of laboratory test and hematoma volumes.

## INTRODUCTION

Spontaneous intracerebral hemorrhage (sICH) accounts for 70% of cases of hemorrhagic stroke, and is associated with high rates of mortality and morbidity [1–3]. Secondary brain injury is thought to result from the development of an inflammatory response [4]. The immune responses developing around the hematoma consist of leukocyte infiltration, microglia activation, and release of various cytokines [5–7]. In addition, a systemic inflammatory response develops, which is reflected by the elevated number of inflammatory cells, such as neutrophils, monocytes, lymphocytes, as well as the presence of inflammatory biomarkers such as C-reactive proteins [8, 9]. Many previous studies reported a role for inflammatory biomarkers in predicting prognosis in sICH. For instance, peripheral blood leucocyte counts were significantly associated with early neurologic deterioration [4, 8] and prognosis [10]. Recently research have indicated that the neutrophil to lymphocyte ratio (NLR) is an independent predictor for clinical outcomes for various cancer types [11, 12], cardiovascular disorders and ischemic stroke [13–16]. The NLR is a reflection of innate (neutrophils) and adaptive (lymphocytes) immune responses. The elevated NLR with increasing neutrophils and lymphocyte depletion indicates the break of immune system balance. The NLR is considered a biomarker for assessing the balance of pro-tumor and anti-tumor inflammation [17], and a higher NLR indicates an increased risk for tumor development [18]. In addition, NLR have been considered a poor prognostic marker in patients with acute coronary syndromes [19] and acute ischemic stroke [20], because NLR reflects recruitment of immune cells and release of inflammatory cytokines. An elevation of peripheral neutrophils increases the damage to the blood brain barrier and neuronal damage, which results in a new phase of inflammatory reaction and further aggravates the damage to the brain tissue [21].

However, the association of NLR and the clinical outcomes in sICH is still up to debate. Previous studies showed that NLR had no significant effect on the mortality [22, 23] and 90-day poor outcomes [23, 24] after multivariate logistic regression. One study [24] showed that an elevated NLR increases the in-hospital mortality in ICH patients rather than 3-month mortality. However, some studies [13, 25, 26] demonstrated that a high NLR was an independent predictor of poor clinical outcomes. Therefore, due to the controversial conclusion, we conducted a meta-analysis to assess the prognostic role of NLR in patients with sICH.

## MATERIALS AND METHODS

### Collect the eligible articles

Online databases PubMed, EMBASE, and Cochrane Central Register of Controlled Trials were queried for papers published from the time of inception of the database to May 1, 2017 using the key words ‘intracerebral hemorrhage’ OR ‘intracranial hemorrhage’ AND ‘neutrophil’ AND ‘lymphocyte’. We reviewed the title and abstract of articles and found the appropriate studies. Additional reports were retrieved by reviewing the references included in the selected articles.

### Include criteria and exclude criteria

We reviewed the titles and abstracts to find the related articles. Then, two reviewers reviewed the full-text of the retrieved articles independently. The articles were included if the met the following inclusion criteria: 1- patients admitted for spontaneous intracerebral hemorrhage; 2- The odds ratio and 95% confidence intervals of mortality or the poor outcomes were provided or could be calculated from the published data; 3- The article being a randomized controlled study (RCTs), cohort or case-control study. Exclusion criteria were: 1- the article is a review, case report, or animal study; 2- The article is not in English; 3- without odds ratio and without related data used to calculate odds ratio.

### Data extraction

Two independent reviewers extracted the data from the included studies and discussed any inconsistencies. Data extraction was standardized based on pre-selected domains including author information, year of publication, country, study design, number of patients, patient demographics such as age and sex, National Institutes of Health Stroke Scale (NIHSS) or Glass coma scale (GCS), hematoma volume, and cut-off value of NLR, study period, odds ratio (OR) and 95% CI on the mortality and 90-day poor outcomes. If the ORs of univariate regression analysis and multivariate regression analysis were available, the values of multivariate regression analysis would be used. The mortality and the poor outcomes were considered the end points. A Rankin Scale (mRS) from 3 to 6 was considered a poor outcome.

### Quality assessment of the selected articles

The quality of included studies was assessed by two reviewers independently with the Newcastle-Ottawa scale criteria [27] for cohort and case-control studies. Studies with Newcastle-Ottawa scale score > 6 were considered high quality studies.

### Data analysis

The data were pooled by the Review Manager Version 5.3 (Cochrane collaboration, Oxford, UK) and publication bias was assessed by STATA 13.0 (STATA Corporation, College Station, TX). The OR and 95% confidence intervals were pooled to analyze the correlation of NLR with mortality and poor outcomes. The fixed-effects model was adopted if *I*^2^ < 50% or *p* > 0.10, otherwise, the random-effects was used. The sensitivity analysis was used to confirm the robustness of the pool outcomes in studies with large heterogeneity [28]. In addition, subgroup analyses was performed to determine the origin of heterogeneity. Publication bias was assessed by Funnel plot with Begg rank correlation. A *p* value < 0.05 was set for statistical significance.

## RESULTS

### Literature research

**Figure 1:**
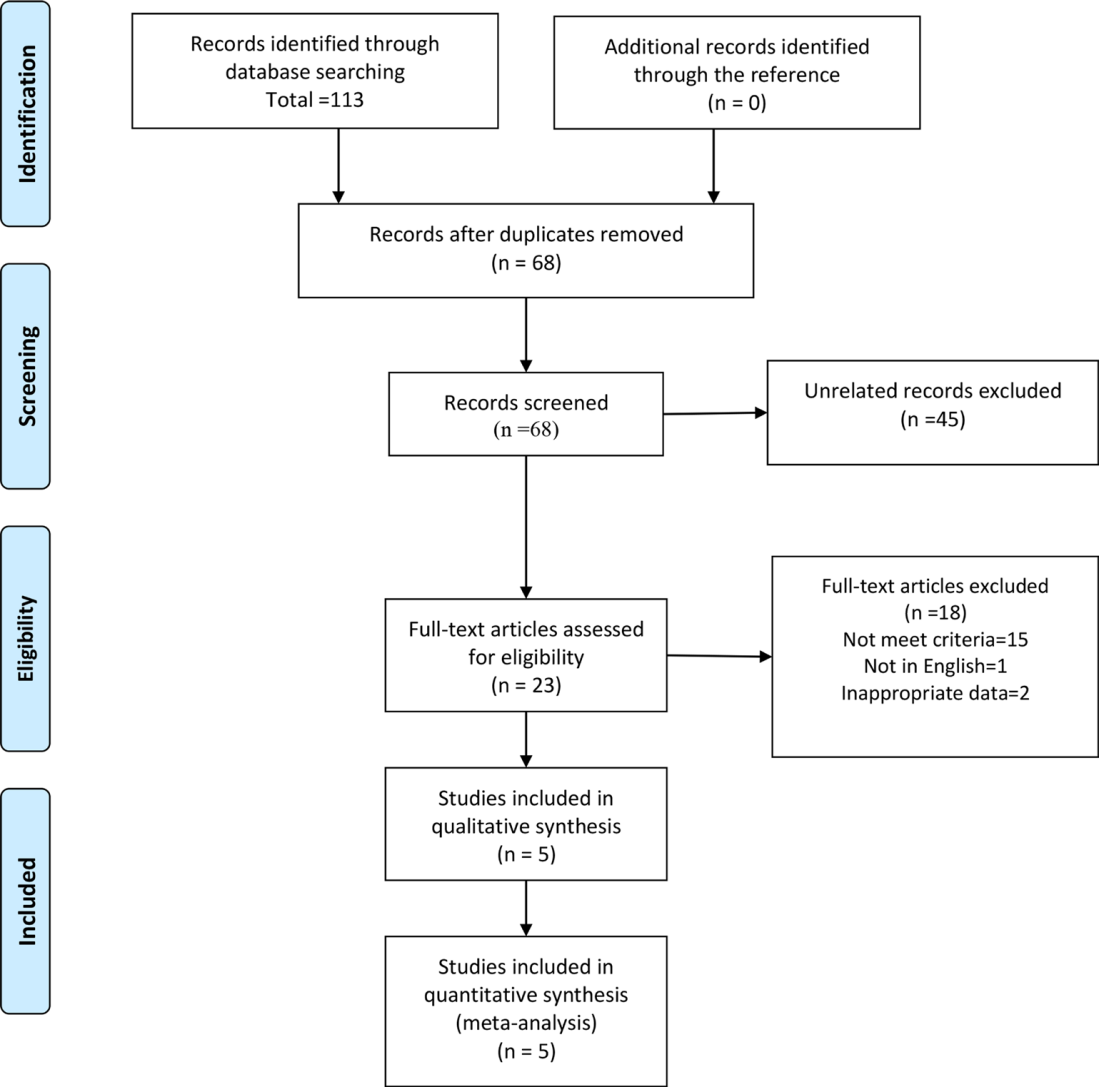
The flow diagram of procedure to search the eligible studies

The studies were searched according to the PRISMA flow diagram [29] (Figure 1). The titles and abstracts of 68 articles were reviewed. Forty-five articles failed to meet the inclusion criteria and were excluded. After assessing the full-text of the remaining 23 articles, 18 articles were excluded for not meeting the inclusion criteria (15), one article was written in Japanese and 2 articles did not include the required data. Five articles were included in the analysis.

### Characteristics of included studies

The data for 1944 subjects were retrieved from the 5 articles included in this meta-analysis. Three articles were conducted in china and two were done Europe. All articles (2 prospective and 3 retrospective) were published between 2016 and 2017. The main cut-off values for NLR ranged from 4.08 to 7.35, and two articles also reported other cut-off values to find the optimal value. All of ORs and 95% CI are based on the multiple regression analysis, except for the OR with cut-off of 8.508 reported by Giede-Jeppe [24]. All included articles were of high quality based on a Newcastle-Ottawa scale score > 6 (Table 1).

**Table 1:** (A) Baseline characteristics of included studies

Author	Year	Country	Study design	Patients (*n*)	Sex (M/F)	Mean age (years)	NIHSS	GCS	Study period	NOS score
Tao, C.	2017	China	Retrospective	336	216/120	58.5	-	11	2010–2013	7
Sun, Y.	2017	China	Prospective	352	234/118	64.2	7.2	-	2011–2014	8
Giede-Jeppe, A.	2017	Germany	Prospective	855	459/396	71.5	14.3	12.2	2006–2014	8
Wang, F.	2016	China	Retrospective	224	141/83	67.9	-	12.6	2012–2014	7
Lattanzi, S.	2016	Italy	Retrospective	177	63/114	67.1	9	-	2008–2015	8

NIHSS: National Institutes of Health Stroke Scale, GCS: Glass coma scale, NOS: Newcastle-Ottawa scale score.

**Table 1:** (B) Baseline characteristics of included studies

Author	Hematoma volume (ml)	Time of laboratory test	Main cut-off value	Other cut-off values	poor outcome (OR)#	mortality (OR)#	Follow up
Tao, C.	15.8	admission	6.62	-	2.6 (1.4–4.7)	5.1 (2.6–8.6)	90-day
Sun, Y.	10.7	non-admission	4.08	7.85	0.93 (0.34–2.57) 1.83 (0.62–5.39)	1.51 (0.34–6.61) 0.64 (0.15–2.76)	90-day 90-day
Giede-Jeppe, A.	14.4	admission	4.66 4.66	8.508	0.983 (0.939–1.029) 2.251 (1.543–3.282)*	0.967 (0.939–0.997) 0.974 (0.945–1.004) 1.626 (1.186–2.231)*	In-hospital 90-day 90-day
Wang, F.	14.9	non-admission admission	7.35 7.35	-	–	1.091 (1.002–1.188) 0.852 (0.608–1.194)	In-hospital In-hospital
Lattanzi, S.	8.1	admission	4.58	-	1.16 (1.01–1.33)	–	90-day

#: multivariate regression analysis, *: univariate regression analysis, OR: odds ratio.

### Overall analysis

### Association of NLR and in-hospital mortality

**Figure 2:**
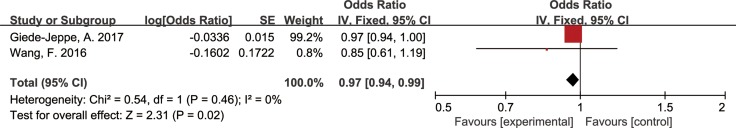
Forest plots for association of NLR and in-hospital mortality

Two articles reporting data from 1079 subjects were pooled to evaluate the relationship between NLR and in-hospital mortality. The result showed that a high NLR significantly increased the in-hospital mortality with OR of 0.97 (95% CI, 0.94–0.99, *p* = 0.02, Figure 2) using a Fixed-effect model. No heterogeneity was detected between the 2 articles (*I*^2^ = 0%, *p* = 0.46).

### Association of NLR and 90-day mortality

**Figure 3:**
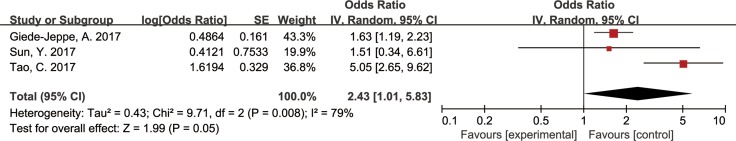
Forest plots for association of NLR and 90-day mortality

Three articles reported the OR and 95% CI of NLR on mortality in 1543 patients. After pooling the data, we found that a high NLR was associated with a higher risk of death with an OR of 2.43 (95% CI, 1.01–5.83, *p* = 0.047, Figure 3) using a Random-effect model. The OR values have no significant change with the exclusion of a single article.

### Association of NLR and 90-day poor outcomes

**Figure 4:**
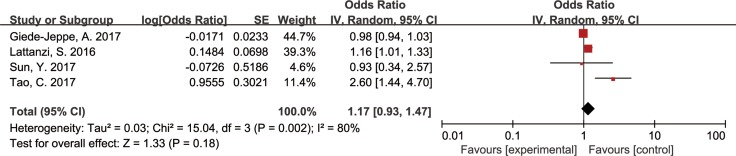
Forest plots for association of NLR and poor outcomes

**Figure 5:**
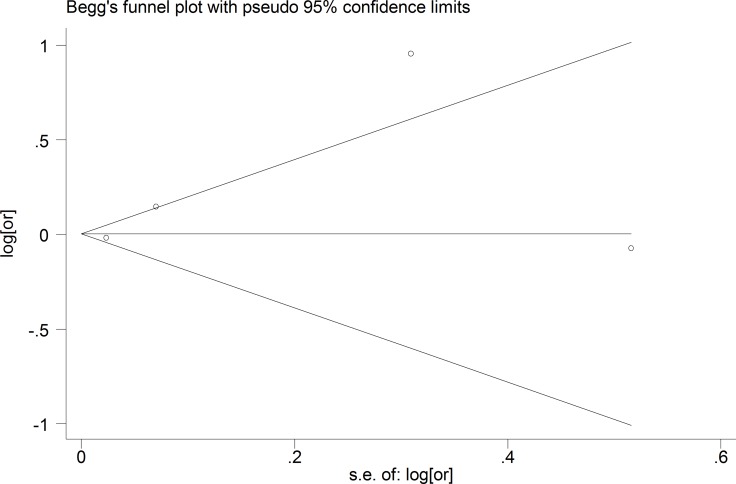
The Begg publication bias plot of the studies reported 90-day poor outcomes, and no publication bias was found in these studies with *P* = 0.734

In four articles covering 1720 patients, the association of NLR and the poor outcomes was assessed using a Random-effect model. Analysis revealed that the NLR had no statistically significant effect on poor outcomes with an OR of 1.17 (95% CI, 0.93–1.47, *p* = 0.18, Figure 4). After the sensitivity analysis, the OR values have no significant change with the exclusion of a single article. (Figure 5).

### Subgroup analyses

The subgroup analyses of mortality (Table 2) and poor outcomes (Table 3) were performed based on countries, time of laboratory test, hematoma volume, cut-off values and duration of follow up. The time of laboratory test was divided into admission group and non-admission group. The admission group included the articles reporting lab data at the time of admission. The articles that collected the blood sample more than 8 hours after admission [22] were included in the non-admission group.

**Table 2:** Subgroup analyses results of 90-days mortality

Groups	*N*	Model	Pooled OR (95% CI)	*p* value	Heterogeneity (*P*, *I*^2^)
Total	3	random	2.43 (1.01–5.83)	0.047	0.008, 79%
China	2	random	3.34 (1.09–10.27)	0.035	0.14, 54%
Europe	1	-	1.63 (1.19–2.23)	0.003	-
Admission NLR	2	random	2.76 (0.91–8.37)	0.07	0.002, 90%
Non-admission NLR	1	-	1.51 (0.34–6.61)	0.58	-
Smaller hematoma	1	-	1.51 (0.34–6.61)	0.58	-
Lager hematoma	2	random	2.76 (0.91–8.37)	0.07	0.002, 90%
Low cut-off value	2	fixed	0.97 (0.95–1.00)	0.09	0.56, 0.0%
Moderate cut-off value	1	-	5.05 (2.65–8.62)	< 0.001	-
High cut-off value	2	fixed	1.56 (1.15–2.13)	0.005	0.22, 33%

N: number of included studies, OR: odds ratio, CI: confidence interval, smaller hematoma: hematoma volume < 14 ml, lager hematoma: hematoma volume > 14 ml. Low cut-off value: 4–5, Moderate cut-off value: 6.5–7.5, High cut-off value: 7.5–8.5.

**Table 3:** Subgroup analyses results of 90-days poor outcomes

Groups	*N*	Model	Pooled OR (95% CI)	*p* value	Heterogeneity (*P*, *I*^2^)
Total	4	random	1.17 (0.93–1.47)	0.18	0.002, 80%
China	2	random	1.70 (0.63–4.58)	0.30	0.09, 66%
Europe	2	random	1.05 (0.90–1.24)	0.52	0.02, 80%
Admission NLR	3	random	1.19 (0.93–1.52)	0.17	< 0.001, 87%
Non-admission NLR	1	-	0.93 (0.34–2.57)	0.89	-
Smaller hematoma	2	fixed	1.16(1.01–1.32)	0.04	0.67, 0.0%
Lager hematoma	2	random	1.53(0.59,3.94)	0.38	0.001, 90%
Low cut-off value	2	random	1.05 (0.91–1.21)	0.51	0.08, 61%
Moderate cut-off value	1	-	2.60 (1.44–4.70)	0.002	-
High cut-off value	2	fixed	2.20 (1.54–3.14)	< 0.001	0.72, 0%

N: number of included studies, OR: odds ratio, CI, confidence interval, smaller hematoma: hematoma volume < 14 ml; lager hematoma: hematoma volume > 14 ml. Low cut-off value: 4–5, Moderate cut-off value: 6–7.5, High cut-off value: 7.5–8.5.

### Subgroup analysis of 90-day mortality

Subgroup analysis based on country revealed that the NLR had significant effect on mortality in China with an OR of 3.34 (95% CI, 1.09–10.27, *p* = 0.035) and in Europe with OR of 1.63 (95% CI, 1.19–2.23, *p* = 0.003). Analysis based on the timing of performing the laboratory tests (admission or non-admission), the pooled OR for mortality were 2.76 (95% CI, 0.91–8.37, *p* = 0.07) and 1.51 (95% CI, 0.34–6.61, p=0.58) respectively. The pooled OR for mortality was 2.76 (95% CI, 0.91–8.37, *p* = 0.07) in larger hematoma and 1.51 (95% CI, 0.34–6.61, *p* = 0.58) in smaller hematoma. Two articles reporting the cut-off values from 4 to 5, the OR for mortality was 0.97 (95% CI, 0.95–1.00, *p* = 0.09). One articles reporting the cut-off values from 6 to 7.5, the OR for mortality was 5.05 (95% CI, 2.65–8.62, *p* < 0.001). The cut-off values from 7.5 to 8.5 assessed in two articles, the OR for mortality was 1.56 (95% CI, 1.15–2.13, *p* = 0.005).

### Subgroup analysis of poor outcomes

The duration of follow up was 90-days in all the articles reporting poor outcomes. The pooled OR for poor outcomes in China was 1.17 (95% CI, 0.93–1.47, *p* = 0.18) and the pooled OR was 1.05 (95% CI, 0.90–1.24, *p* = 0.52) in Europe. In patients with laboratory test performed at the time of admission, the pooled OR for poor outcome was 1.19 (95% CI, 0.93–1.52, *p* = 0.17), and the pooled OR was 0.93 (95% CI, 0.34–2.57, *p* = 0.89) in the patients having test within 24h. The pooled OR for poor outcome in smaller hematoma was 1.16 (95% CI, 1.01–1.32, *p* = 0.04), while the pooled OR for poor outcome in larger hematoma was 1.53 (95% CI, 0.59–3.94, *p* = 0.38). Two articles reporting the cut-off values from 4 to 5, the OR for poor outcome was 1.05 (95% CI, 0.91–1.21, *p* = 0.51). One article reporting the cut-off values from 6 to 7.5, the OR for poor outcome was 2.60 (95% CI, 1.44–4.70, *p* = 0.002). The cut-off values from 7.5 to 8.5 assessed in two articles, the OR for poor outcome was 2.20 (95% CI, 1.54–3.14, *p* < 0.001).

### publication bias

**Figure 6:**
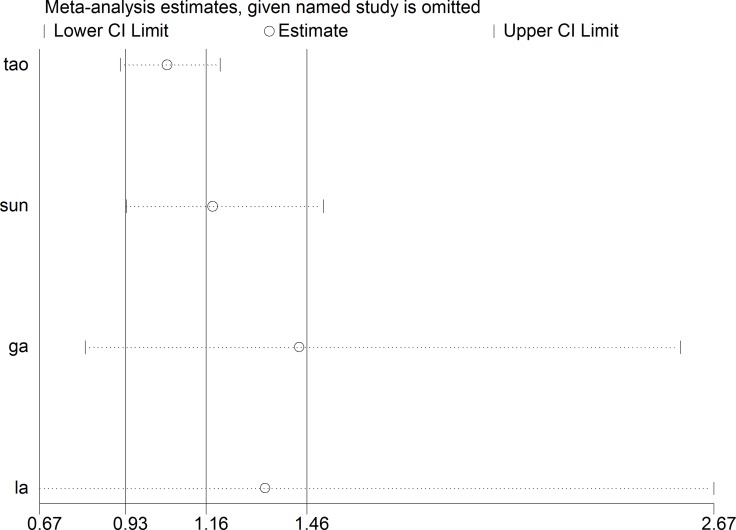
The sensitivity analysis of the studies reported 90-day poor outcomes and the outcomes had no significant change after excluding a single study

The publication bias was assessed by STATA 13.0 and no publication bias was found for 90-day poor outcomes (*P* = 0.734) in this meta-analysis (Figure 6).

## DISCUSSION

### Implications

Inflammation is considered a major player in the development of secondary brain injury after stroke. Tao et al. [13] demonstrated that a higher white blood cell, neutrophil count and NLR were independent predictors for 90-day poor outcomes after sICH, the predictive ability of NLR in sICH remains controversial. To the best of our knowledge, this is the first study performing a meta-analysis to systematically evaluate the association of NLR and clinical outcomes of sICH. In the present study, we found that higher NLR was associated with higher rates of in-hospital mortality and 90-day mortality in sICH. In patients with a small hematoma or a high cut-off value, the high NLR had a statistically significant association with poor outcomes (mRS ≥ 3) in sICH.

NLR is considered a comprehensive index that reflects the degree of inflammation infiltration (neutrophil) [30–32], and immune status (lymphocytes) [32]. The activation of inflammatory cells, cytokines results in BBB disruption, tissue damage, and massive neuronal cell death [7, 21]. Peripheral blood is also used as a biomarker for systemic inflammatory response. This was found to be associated with the clinical and radiological severity of sICH [33]. In addition, lymphocytes induce cellular and humoral immune responses, which play an important role in host defense against pathogens. Reduced lymphocyte counts in lymphoid organs and peripheral blood renders patients more prone to infections [32, 27], which plays an important role in the prognosis after sICH [34]. We found the NLR was associated with the mortality of sICH, which was attributed to the increase of peri-hematoma or systemic inflammatory response and ICH-induced immunosuppression.

The cut-off values for various diseases were different, for instance, the value was from 4.1 to 5.9 in the ischemic stroke [15, 16] and from 3.5 to 7.6 in cardiovascular diseases [14]. In patients with sICH, the main cut-off value ranged from 4.08 to 7.35 in the available articles. Three of the included articles suggested the values were about 4.5, while Wang [22] and Tao [13] reported cut-off values of more than 6. The cut-off values could be affected by many causes, such as different biochemical analyzers, time of blood sample, capacity of immune system, and hematoma volume. We found that higher cut-off values had a better predictive ability of NLR for the prognosis. Sun [23] reported that high NLR was not associated with poor outcome or mortality with a cut-off value of 4.08. Giede-Jeppe [24] showed the high NLR was an independent predictor for poor outcomes and 90-day mortality with a high cut-off value of 8.508. In addition, Tao [13] demonstrated the NLR had a high specificity of 0.767 for 90-day mortality with a relative high cut-off value. Wang [22] also suggested aNLR with cut-off of 7.35 had a significant association with the in-hospital mortality.

Hematoma volume is known as an independent predictor for clinical outcomes in sICH [8]. Some studies [35, 36] showed that higher peripheral WBC was found in patients with larger hematoma volumes and Sun [23] demonstrated that patients with a higher NLR at the time of admission had larger hematoma volumes. The mechanism is most likely be related to a stress-induced response [35] or an inflammatory response in modulating the coagulation cascade after sICH [36]. With larger hematomas, the cut-off values might be affected by the increasing of NLR. In this meta-analysis, we found that cut-off values increased with the increasing of hematoma volume (Table 1). Tao [13] and Wang [22] included patients with a larger mean hematoma volume, and demonstrated higher cut-off values NLR when compared to the other articles. Thus, a lower cut of value < 4.5 have been suggested for smaller hematomas, while higher cut-off values are suggested for larger hematomas. NLR with cut-off values of 7.5 could predict the mortality and poor outcomes, regardless of other factors, such as hematoma volumes and admission NLR. However, the cut-off values should be set according to mean hematoma volume data established in different medical institutions.

The NLR of sICH is time dependent and values increase with time during the first few days [24, 37]. Animal experiments revealed that neutrophil infiltration, which enhances the peri-hematoma edema develops within 24 hours from the onset of ICH, and reaches a maximum 3 days from onset [30–32]. The reduction of lymphocytes in lymphoid organs and peripheral blood occurs within 12 h [37]. In a clinical trial, Giede-Jeppe [24] found that NLR 1 day after admission was significantly higher than NLR at the time of admission, and that NLR reached peak levels after 2 days. They found a statistically significant association between NLR at the time of admission and outcome; however, they did not discuss the predictive ability of NLR at 2 or 3 days. Wang [22] compared the predictive ability of NLR at admission with the NLR on the next day. They found that next day NLR was associated with a significant predictive potential for in hospital mortality, rather than the NLR at admission. Based on the analysis conducted in this report, no difference in predictive potential was detected between admission and non-admission NLR. Further investigations are required to determine the role of time of acquiring NLR on outcomes.

The clinical outcomes were affected by various factors other than NLR, such as blood pressure [38, 39], blood glucose level [40], temperature [41], anticoagulant drugs [42] and some other factors [43–45] related to metabolic homeostasis, inflammatory responses, and perfusion disturbances. Thus, a multidimensional evaluation is essential for the appropriate management of sICH. Future treatments should develop based on the proper understanding of stroke pathophysiology and course. These factors could be targeted for the treatment of sICH, and the better understanding of the interplay of these factors can help produce more efficient prognostic algorithms.

However, significant heterogeneity exists among the five studies. The main reasons were the hematoma volume, time of laboratory test and cut-off values. The mean hematoma volume ranged from 8.1 to 15.8ml in the included articles. After excluding the two articles with larger hematomas, we found the heterogeneity to be 0.0% in smaller hematomas (Table 3). Two articles [22, 23] with non-admission NLR also had no heterogeneity for predicting the mortality (Table 2). The heterogeneity was not significant in the articles with high cut-off values (Table 2 and Table 3). In addition, Wang [22] demonstrated the negative effect of NLR on short-term mortality, and the incidence of death was lower than the other articles with a longer period of follow-up.

### Limitations

There were some limitation in this meta-analysis. First, the prognostic role of NLR in the ICH was discussed with the limited number of articles and subjects. Second, the heterogeneity between articles was significant, and the random-effects model was adopted. Third, the main cut-off values were different in the included articles. We did the subgroup analyses to find the origin of heterogeneity to estimate the results more accurately.

### Future study

More high-quality research with larger sample size is needed to identify the association between NLR and clinical outcomes in sICH, considering the time point of blood sampling, time from onset, and long-term or short-term follow up. Moreover, future studies should consider some other factors which are associated with the outcomes of sICH patients, such as hematoma volumes, blood pressure, blood glucose level, temperature, and history of anticoagulant drugs. With intervention to local or systemic inflammatory response, the change of NLR and the predictive ability of NLR for outcomes in sICH needed more study.

## CONCLUSIONS

The results of this meta-analysis demonstrated that the high NLR was a predictor of the 90-day mortality and in-hospital mortality in patients with sICH, but not a predictor of poor outcomes. The NLR with cut-off values of 4.5 could predict the poor outcomes in patients with smaller volume hematomas, while the NLR with cut-off values of 7.5 had a significant predictive ability for mortality and poor outcomes, regardless of country, time of laboratory test and hematoma volume.
